# Exploring ultrasound combined with *Litsea pungens Hemsl* essential oil compound coating for modulating flavor and metabolites in Nuodeng ham: A multidimensional analysis by UHPLC-QE-MS and GC-IMS

**DOI:** 10.1016/j.fochx.2025.103404

**Published:** 2025-12-11

**Authors:** Nannan Zhou, Lihong Zhang, Guiying Wang, Ruwei Ren, Yanfei Du, Shuai Tang, Jiayan Tan, Guozhou Liao

**Affiliations:** a College of Food Science and Technology, Yunnan Agricultural University, Kunming 650201, China; b Livestock Product Processing and Engineering Technology Research Center of Yunnan Province, Yunnan Agricultural University, Kunming 650201, China

**Keywords:** Nuodeng ham, *Litsea pungens Hemsl* essential oil, Ultrasonication, Volatilomics, Metabolomics

## Abstract

The mechanism of ultrasound (US) combined with *Litsea pungens Hemsl* essential oil (LO) compound coating on Nuodeng ham was investigated at defined power levels, including LO (0 W), L-US (low, 200 W), M-US (middle, 400 W), and H-US (high, 600 W). The differences in color, texture, volatile flavor substances, and metabolic networks among treatment groups were resolved by GC-IMS and UHPLC-QE-MS. The results indicated that both L* and a* values in the LO group were notably higher than those of the control group (CG), whereas the H-US group showed a significant reduction in hardness. The flavor analysis revealed that the compound treatment enhanced the flavor substances, including hexanal and 2-heptanone. A total of 67 differential metabolites were identified, confirming that the treatment promoted the accumulation of flavor metabolites through the regulation of amino acid metabolism, TCA cycle, and lipid metabolism. These results provide a theoretical basis for fermented meat processing.

## Highlights

• LO and L-US could improve the color and sensory characteristics of ham.
• LO and M-US promoted the formation of flavor substances such as hexanal.
• A total of 32 characteristic flavor markers and 67 differential metabolites were identified.
• Amino acid and lipid metabolism were the key metabolic pathways of the Nuodeng ham.
• The combination of 400 W ultrasound and LO treatment yielded the optimal result.

## 1 Introduction

Nuodeng ham is one of the representative hams in Yunnan Province, produced in Nuodeng Village, a thousand-year-old salt village in western Yunnan. It is cured with locally produced well salt. Because it is covered with a layer of Nuodeng ancient salt well brine mud, also known as “Salt mud ham”, it is popular for its mellow aroma and bright color (Liu et al., 2019; Zhou et al., 2025). Traditional Nuodeng hams are usually stacked or hung during the long-term storage phase. The direct exposure of the hams to the air makes the natural pigment components of hams susceptible to oxidation, resulting in a darker appearance. Meanwhile, it also affects the texture and salt content of ham, ultimately affecting the overall quality of the product and consumer acceptance (Zhou et al., 2019). In addition, during the post-ripening and storage stages of ham, numerous diverse biochemical reactions, represented by lipid and protein oxidation will occur, thereby causing adverse influences on the quality of the ham, such as flavor attenuation and lack of aroma (Zhang et al., 2020; Zhou et al., 2025). These problems have largely limited the development of the Nuodeng ham industry and affected its economic benefits.

In recent years, the application of edible films and protective coatings in meat products has gradually become a research hotspot due to their natural, non-toxic, and degradable advantages (Kupervaser et al., 2023). Studies have shown that these macromolecular coatings can effectively inhibit moisture loss, oxidation, discoloration, and microbial contamination of meat products, as well as reduce nutrient depletion (Catarino et al., 2017). Chitosan, as the only alkaline polysaccharide in nature at present, is considered to be an ideal biopolymer for the production of active edible coatings due to its good biodegradability, biocompatibility, antimicrobial activity, antioxidant, and non-toxicity (Bie et al., 2013; Feng et al., 2016). In addition, it has been reported that active compounds such as plant essential oils, nanomaterials, herbal and spice extracts, and probiotics can be incorporated to not only expand the versatility and utility of edible films but also to improve food quality (Umaraw et al., 2020). Studies have shown that when chitosan is coated with plant essential oil, plant essential oil can be effectively embedded, thus playing a role in controlling the sustained release and reducing the adverse effects of plant essential oil on product flavor (Ju et al., 2019), which has also been confirmed in previous studies of our team (Zhang et al., 2022). Researchers investigated the flavor quality of chilled fresh mutton by using cumin essential oil, *Zanthoxylum* essential oil, and a blend of the two essential oils, and the findings demonstrated that the blended essential oil group was able to increase the content of volatile flavor compounds and improve the overall flavor of chilled fresh mutton (Li et al., 2023). Bi et al. (2019) applied a compound coating of black pepper essential oil to Jinhua ham and found that the aromatic content of black pepper essential oil significantly increased in the treatment group after 4 months of storage, improving the overall aroma score of Jinhua ham.

In recent years, ultrasonic technology has been more and more widely used in meat processing. This technology has the advantages of promoting the penetration of excipients in meat and meat products, promoting the texture characteristics of meat products, and prolonging the shelf life (Wang et al., 2018). Evidence from studies has shown that ultrasonic treatment positively affects the inhibition of protease and lipase activity, enabling the modulation of the liberation process of amino acids and fatty acids, and contributing to the extraction of flavoring substances from fragrances (Teng et al., 2019). Zhou et al. (2021) demonstrated that ultrasound-assisted heating significantly improved the sensory and aroma profiles of defective Jinhua ham, a change attributed to an increase in fruity compounds and a decrease in acidic compounds. Meanwhile, Zhang, Xing, et al. (2021); Zhang, Zhang, and Xing (2021) identified nine key metabolites responsible for enhancing the taste of unsmoked bacon. Furthermore, Alves et al. (2020) confirmed that ultrasound influences proteolysis and lipid oxidation, thereby regulating the formation of free amino acids and volatile flavor compounds in dry-fermented sausages.

*Litsea pungens Hemsl* (LpH), also known as *Litsea cubeba*, produces an essential oil that is one of its active secondary metabolites. This oil is primarily composed of terpenes, alkenes, alcohols, phenols, aldehydes, ketones, esters, and other complex hydrocarbons, and it functions to inhibit spoilage and pathogenic microorganisms, maintain food stability during storage, and prevent oxidation and discoloration (Liu et al., 2024). Therefore, the essential oil of LpH demonstrates a better application prospect in the meat processing industry. However, the effect of ultrasonic treatment combined with compound essential oil coating on the flavor quality of dry-cured ham has not been reported.

Given this, to solve the problem of oxidative discoloration and flavor degradation of Nuodeng ham during storage, the present study utilized ultrasonic treatment with different powers (0 W, 200 W, 400 W, 600 W) combined with *Litsea pungens Hemsl* essential oil (LO) compound coating to treat the cut pieces of Nuodeng ham. After three months of storage, changes in ham color, texture, volatile flavors, and small molecule metabolites were analyzed using GC-IMS and UHPLC-QE-MS. This investigation aimed to elucidate the mechanism by which the treatment influences the flavor quality of Nuodeng ham during storage, thereby providing a theoretical basis for its quality preservation and for the application of compound essential oil coatings on dry-cured ham.

## 2 Materials and methods

### 2.1 Design of experiment

Preparation of *Litsea pungens Hemsl* essential oil compound: A chitosan coating solution was prepared by dissolving 2 % chitosan in acetic acid (1 %) in a water bath at 45 °C. After complete dissolution, add glycerol (1 mL of glycerol per 100 mL of chitosan coating solution) as a plasticizer, and then add 0.5 % of the essential oil of wood ginger (purity ≥95 %) to obtain the *Litsea pungens Hemsl* essential oil compound (Zhang et al., 2022).

The five hams, processed from the same batch and age group of Nuodeng local black pigs, were purchased from Yunnan Ruitong Animal Husbandry Science and Technology Development Co., Ltd., a certified enterprise in breeding, slaughtering, and processing. Its operations comply with national food safety regulations and animal welfare standards. The biceps femoris muscle was selected as the test sample, cut into 30 small pieces (approximately 10 cm × 10 cm each), and randomly allocated into 5 groups of 6 pieces per group. They are the control group (CG, vacuum-packed ham after cutting), *Litsea pungens Hemsl* essential oil compound coating group (LO, the prepared *Litsea pungens Hemsl* essential oil compound was evenly coated on the surface of the cut ham, formed a film and then vacuum-packed), low-power ultrasound combined with *Litsea pungens Hemsl* essential oil compound coating group (L-US, the *Litsea pungens Hemsl* essential oil compound was uniformly applied to the surface of the ham, allowed to form a film and then vacuum-packed, followed by 200 W ultrasonication for 5 min), medium-power ultrasound combined with *Litsea pungens Hemsl* essential oil compound coating group (M-US, the *Litsea pungens Hemsl* essential oil compound was uniformly applied to the surface of the ham, allowed to form a film and then vacuum-packed, followed by 400 W ultrasonication for 5 min), and high power ultrasound (H-US, the *Litsea pungens Hemsl* essential oil compound was uniformly applied to the surface of the ham, allowed to form a film, and then vacuum-packed, followed by ultrasonication at 600 W for 5 min). Ultrasonication was performed using a KQ-600VDV unit (Kunshan, China). All treatments were conducted in an ultrapure water medium under fixed conditions of 20 kHz frequency and a temperature of 25 ± 1 °C. The treated groups of hams were stored at room temperature for 3 months and then sampled for analysis.

### 2.2 Color determination

Using a colorimeter, the color parameters (CS10, Shanghai, China) of the ham were measured at three randomly selected points within the lean tissue after sample collection. The lightness (L*), redness (a*), and yellowness (b*) values of the tested samples were then recorded and documented. Three repeated measurements were performed for each point.

### 2.3 Texture determination

Texture profile analysis (TPA) was conducted using a P/36R probe, with meat samples from each group cut into 2 cm^3^ cubic specimens along the muscle fiber direction. The trigger was set to Auto (Force) mode with a trigger force of 5.0 g. The probe speeds were 2.00 mm/s for the pre-measurement, 1.00 mm/s during the measurement, and 1.00 mm/s for the post-measurement, with a strain of 50.0 %. Each sample was measured in triplicate. Furthermore, the tenderness of the samples was determined by employing an HDP/BSW probe under the Compression testing mode. The instrumental settings for tenderness determination were configured based on the prior research findings of our research team (Li, Zheng, et al., 2024).

### 2.4 Sensory evaluation

Sensory evaluation procedures, sample treatment methods and sensory scoring criteria were referred to the previous methods of the research team (Li, Zheng, et al., 2024). It's worth noting that sensory experiments at Yunnan Agricultural University are exempt from Human Ethics Committee approval; additionally, the university lacks formal documentation protocols for sensory research. All participants provided written informed consent acknowledging potential risks and their right to withdraw at any time. The consent forms contained explicit privacy clauses, and all personal information underwent anonymization and encryption. Data access was limited to authorized personnel, and participants retained unconditional withdrawal rights without penalty. The study cohort comprised 20 certified evaluators (10 male, 10 female; mean age 25.5) from the Food Science Department, each with >1 year of sensory assessment experience. Participants were screened for the absence of olfactory/gustatory impairments and good health. Following training per Chinese National Standard GB/T 16291.1–2012, all demonstrated proficiency in identifying the five basic taste modalities.

### 2.5 Determination of volatile flavor compounds

The volatile flavor profile of Nuodeng ham was systematically analyzed through gas chromatography-ion mobility spectrometry (GC-IMS) following standardized analytical protocols. Accurately measured 2 g sample portions were hermetically sealed in 20 mL headspace vials, followed by thermal treatment at 60 °C for a 20 min duration to expedite the volatilization of target analytes. The liberated headspace components were subsequently analyzed using a FlavourSpec® GC-IMS system under optimized parameters. With the help of the special analysis software developed by the G.A.S. company, the characteristic difference spectra of volatile organic compounds in samples can be obtained. The software integrates the NIST standard database and IMS database to achieve accurate qualitative identification of volatile compounds. Detailed parameters of GC-IMS analysis and detection are shown in Table S1.

### 2.6 Determination of small molecule metabolites by UHPLC-QE-MS

The freeze-dried ham samples were weighed at 50 mg and portioned into 1.5 mL microcentrifuge tubes. An aliquot of 400 μL of pre-cooled organic extractant (acetonitrile-methanol, 1:1, *v*/v) was added, and the extraction was assisted by low-temperature ultrasonication (5 °C, 40 kHz, 0.5 h) after vortexing for 30 s. The extraction system was allowed to stand at −20 °C for 0.5 h, and then the solid-liquid separation was achieved by centrifugation at 13,000 ×*g* for 15 min at 4 °C. After the supernatant was concentrated and dried under nitrogen, and 120 μL of re-solvent (acetonitrile-water, 1:1, v/v) was used for gradient re-solubilization, and the system was subjected to a second low-temperature ultrasonication treatment (5 °C, 40 kHz, 5 min), and the clarified liquid was centrifuged at 13,000 ×*g* for 5 min, and then transferred to the vials with an internal cannula for chromatography analysis. The quality control system was established simultaneously, and the batch QC samples were prepared by combining the aliquots of each sample (20 μL/sample), and the instrumental detection was carried out in parallel.

LC-MS/MS analysis was performed using an ultra-high performance liquid chromatography tandem Fourier transform mass spectrometry (UHPLC-Q Exactive -HFX) system from Thermo Fisher Scientific (USA). The specific chromatographic and mass spectrometric condition parameters are described in Table S2.

### 2.7 Statistical analysis

Each group of experimental samples was repeated at least 3 times. SPSS software (V 17.3, USA) was utilized to perform statistical analyses based on calculated mean values and corresponding standard deviations. Significant differences among group means were identified using Duncan's multiple range test following one-way analysis of variance (ANOVA), with statistical significance established at *P* < 0.05.

Volatile compound data from different treatment groups of Nuodeng ham were collected and analyzed using analytical software, including LAV (Laboratory Analytical Viewer) with its plugins (Reporter, Gallery plot), and the GC-IMS Library Search qualitative analysis tool. PCA and PLS-DA were conducted using SIMCA-P14.1, while metabolomics data analysis and heatmap clustering were performed via MetaboAnalyst 5.0. The metabolic pathways associated with the compounds were analyzed by the Kyoto Encyclopedia of Genes and Genomes (KEGG) biological pathway repository (https://www.genome.jp/kegg/).

## 3 Results and discussion

### 3.1 Analysis of color results

Color serves as a critical indicator in the quality assessment of meat products, directly affecting consumers' willingness to purchase and their sensory experience. In this study, the LO group of hams had the highest L* value of 44.83 (Table 1), which was significantly higher than the CG, L-US, M-US, and H-US groups by 9.74 % (*P* < 0.05), 4.55 % (*P* < 0.05), 2.16 %, and 4.99 % (*P* < 0.05), respectively. Meanwhile, the a* value of ham in the LO group was also the highest at 9.94, which was considerably higher than that of the CG and ultrasound-treated groups. Specifically, compared with the CG, L-US group, M-US group, and H-US group, the a* value in the LO group was markedly increased by 33.96 % (*P* < 0.05), 15.72 % (*P* < 0.05), 22.56 % (*P* < 0.05), and 24.25 % (*P* < 0.05), respectively. The pigment in ham is mainly nitrosomyoglobin, which gives ham its bright rose-red color. Studies have shown that some reducing substances, such as sodium isoascorbate, can stabilize nitrosomyoglobin (Suman et al., 2016). LO is rich in terpenes and has a strong antioxidant effect, can stabilize nitrosomyoglobin, and contribute to enhancing the color of ham (Bao et al., 2023; Zheng et al., 2018). In contrast, the LO group exhibited the lowest b* values, while the control group demonstrated the highest b* values, followed by the ultrasonic treatment groups in this work. Notably, the a* values in the ultrasonic treatment groups (L-US, M-US, H-US) were lower than those in the LO group but significantly higher than those in the CG group (*P* < 0.05). Simultaneously, compared with the CG group, these treatments significantly reduced the b* values. This result demonstrates the potential of this treatment to effectively delay the deterioration of ham color. This phenomenon may be attributed to the cavitation effect of ultrasound, which facilitates the penetration of bioactive components from the essential oil into the cut surface of the ham, thereby enhancing their interaction efficiency with myoglobin (Peña-Gonzalez et al., 2019). As a result, the a*value in the ultrasonic treatment groups was significantly higher than that in the untreated CG group. Previous research has indicated that ultrasonic treatment modulates the conformational structure and oxidation pathways of myoglobin in meat products through integrated physicochemical mechanisms, inducing alterations in color parameters. Nevertheless, its influence on the overall chromatic profile remains marginal (Cao et al., 2021). In summary, these findings indicate that LO and ultrasound combined with LO treatment hold potential for improving the color characteristics of ham.

**Table 1:** Effects of different power ultrasound combined with *Litsea pungens Hemsl* essential oil compound coating treatment on the color and texture of Nuodeng ham.

Index	CG	LO	L-US	M-US	H-US
L*	40.85 ± 1.06^c^	44.83 ± 1.33^a^	42.88 ± 1.39^b^	43.88 ± 1.57^ab^	42.70 ± 1.53^b^
a*	7.42 ± 0.34^b^	9.94 ± 0.24^a^	8.59 ± 0.94^b^	8.11 ± 0.97^b^	8.00 ± 0.65^b^
b*	6.69 ± 0.30^a^	4.41 ± 0.98^c^	5.72 ± 0.47^b^	5.84 ± 0.51^b^	5.83 ± 0.46^b^
Hardness (g)	32,401.48 ± 1614.48^a^	30,795.75 ± 2923.66^ab^	30,480.25 ± 1657.74^ab^	28,384.51 ± 1844.51^ab^	27,695.41 ± 2796.19^b^
Springiness	0.47 ± 0.11^a^	0.50 ± 0.08^a^	0.50 ± 0.04^a^	0.52 ± 0.03^a^	0.52 ± 0.06^a^
Cohesiveness	0.41 ± 0.13^a^	0.43 ± 0.03^a^	0.44 ± 0.05^a^	0.43 ± 0.08^a^	0.51 ± 0.07^a^
Chewiness	6464.80 ± 902.79^a^	6613.78 ± 975.00^a^	6697.93 ± 810.12^a^	6322.49 ± 677.53^a^	7350.52 ± 1699.43^a^
Resilience	0.10 ± 0.03^a^	0.13 ± 0.04^a^	0.23 ± 0.04^a^	0.12 ± 0.05^a^	0.14 ± 0.01^a^

Note: In the same row, data with different lowercase letters indicate significant differences (*P* < 0.05).

### 3.2 Texture analysis

Texture, as a core quality attribute of meat products, influences sensory acceptability, processing adaptability and nutritional function through physical properties such as hardness and elasticity (Schreuders et al., 2021). As displayed in Table 1, the hardness of Nuodeng ham decreased with increasing ultrasound power, and the hardness of the H-US group was 27,695.41 g, which was notably lower than the other groups (*P* < 0.05). This may be attributed to the mechanical fracture effect of ultrasonication, which triggers the destruction of the cellular structure of the muscle tissue through cavitation, leading to the dissociation or degradation of myofibrillar and smooth muscle protein structures, thus optimizing the textural properties of the ham (Wang et al., 2020). In addition, ultrasonic treatment enhances meat water-holding capacity by facilitating the migration and homogeneous distribution of water molecules within the muscle tissue, resulting in a reduction in the hardness of meat products (Cao et al., 2021). Notably, other textural parameters, including elasticity, cohesiveness, chewiness, and resilience, exhibited no significant differences among groups, indicating that ultrasound combined with essential oil coating treatment specifically influenced the hardness of Nuodeng ham while exerting no notable impacts on other textural attributes.

### 3.3 Sensory evaluation analysis of Nuodeng ham

**Fig. 1:**
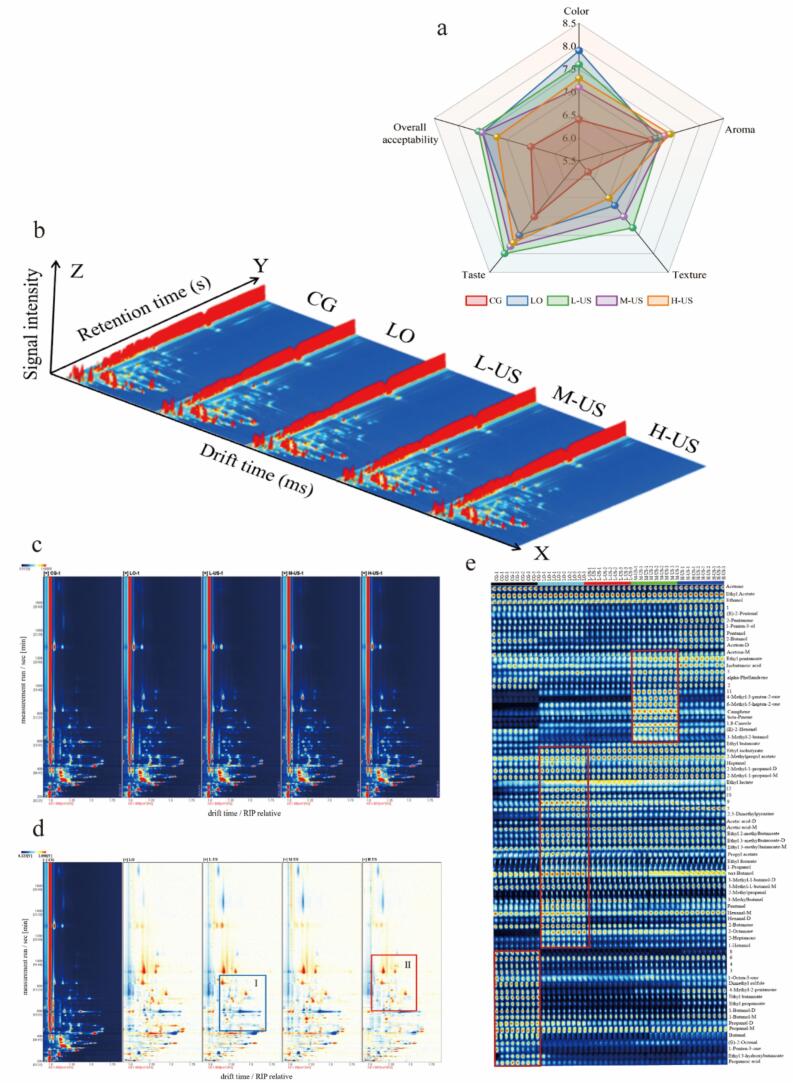
Qualitative analysis of volatile flavor compounds in Nuodeng ham by GC-IMS. (a) Sensory evaluation analysis of Nuodeng ham; (b) Three-dimensional spectrum of volatile flavor components; (c) Two-dimensional map of volatile flavor compounds; (d) Two-dimensional difference map of volatile flavor compounds; (e) Fingerprints of volatile compounds of different treatment groups.

The results of the sensory evaluation showed that there were differences in the sensory attributes of the hams in each group after the ultrasound treatment combined with the essential oil coating (Fig. 1a). The LO group showed the best color characteristics, and its score showed a significant increase compared to the CG group (*P* < 0.05), but the ultrasonic treatment resulted in a significant decrease in this index. Low/medium power ultrasound combined with essential oil coating treatment can significantly improve the taste characteristics of ham, and the L-US group performs best in the texture index. The overall acceptability analysis showed that the LO, L-US, and M-US groups had the highest comprehensive scores, while the 600 W high-power ultrasound treatment group had a marked reduction in this index (*P* < 0.05), indicating that excessively high-power ultrasound would exert an adverse impact on the sensory quality of ham.

### 3.4 Analysis of volatile flavor compounds

#### 3.4.1 Qualitative analysis of volatile flavor compounds

HS-GC-IMS was applied to characterize and identify the volatile flavor compounds in Nuodeng ham treated with different power ultrasound combined with the *Litsea pungens Hemsl* essential oil compound. As depicted in Fig. 1b, through comparative analysis of retention times, migration times, and peak intensities for volatile flavor compounds in each ham group, it can be seen from the three-dimensional map of GC-IMS analysis that there are differences in volatile flavor compounds among the groups. In addition, the two-dimensional spectrum (Fig. 1c) results showed that most of the signal peaks were mainly concentrated in the 0–1000 s range, and the drift time was in the range of 1.0–1.75 s. The peak signal was significantly enhanced in the Nuodeng ham treatment groups. To analyze the volatile flavor compounds differences among all groups of Nuodeng ham, the GC-IMS map was processed using differential comparison: the control group served as the reference, and signal peaks were subtracted to generate a difference map. In the map, white regions indicate no significant difference in substance concentration, blue regions show lower concentrations than the control group, while red regions denote higher concentrations–darker coloration corresponds to a larger difference. As illustrated in Fig. 1d, notable variations in volatile substances existed across treatment groups, where the concentration of substances in region I was reduced and that in region II was elevated. These results indicate that ultrasound at different power levels combined with essential oil coating treatment affects the volatile substances in Nuodeng ham.

During the prolonged fermentation and maturation of ham, the combined action of microbial activities and environmental factors drives the degradation of proteins, lipids, and carbohydrates, generating a diverse array of volatile flavor compounds. The specific composition and concentrations of these substances collectively determine the unique flavor profile characteristic of ham (Petričević et al., 2018). To better observe the effect of ultrasound combined with the *Litsea pungens Hemsl* essential oil compound treatment on the flavor of Nuodeng ham, the volatile flavor substances were qualitatively analyzed. A total of 62 volatile flavor compounds were identified (Table S3) in the ham samples of each group, mainly comprising 12 aldehydes, 15 alcohols, 12 ketones, 4 acids, 14 esters, 3 terpenes, 1 pyrazine, and 1 thioether. Fig. 1e shows the fingerprint of volatile flavor compounds in each group of hams to understand the trend of changes in specific compounds. It can be seen that propionic acid, ethyl 3-hydroxybutanoate, 1-penten-3-one, (E)-2-hexenal, butanal, propanal, and 1-butanol were higher in the CG group; 1-hexanol, 2-heptanone, 2-octanone, 2-butanone, and hexanal were higher in the LO group; ehyl lactate, 2-mthylpropyl acetate, and ethyl isobutyrate were higher in the L-US group; 2-methyl-1-propanol, heptanal, ethyl hexanoate, and 3-methyl-2-butanol were higher in the M-US group; and α-phellandrene, isobutanoic acid, ethyl pentanoate, 2-butanol, and pentanol were higher in the H-US group.

#### 3.4.2 Multivariate statistical analysis of volatile flavor compounds

**Fig. 2:**
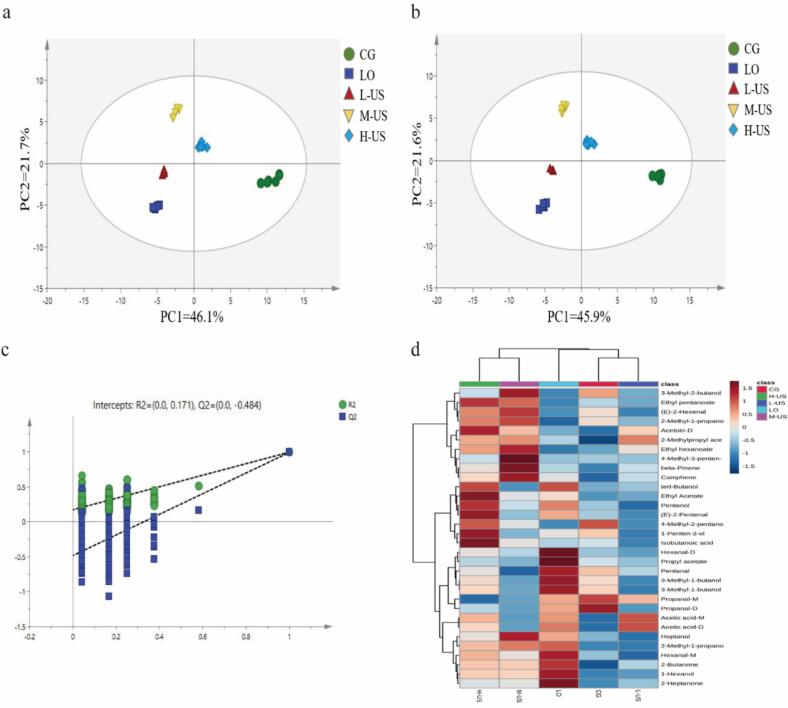
Multivariate statistical analysis of volatile flavor compounds. (a) PCA score plot; (b) PLS-DA score plot; (c) The permutation test diagram; (d) Cluster heat map analysis of flavor characteristic markers of Nuodeng ham.

Fig. 2a depicts a plot of the PCA analysis scores of the signal peak intensities of ham volatile compounds for each test group. All samples were within the 95 % confidence interval, with the first principal component (PC1) explaining 46 % of the variance and the second principal component (PC2) accounting for 22 %, and the cumulative contribution rate of the two amounted to 68 %, which indicated that the extracted principal components were able to effectively explain the information of the original variables. From the spatial distribution characteristics of the sample points, the samples of Nuodeng hams treated with different power ultrasound combined with the compound coating of essential oils of LO showed an obvious trend of separation between groups, reflecting that there were significant differences in the volatile flavor components among the hams of each group under different treatment conditions.

To further elucidate the distinct contributions of flavor compounds across ham groups, PLS-DA was employed for analysis. As depicted in Fig. 2b, samples from each group exhibited differentiated distributions, with model parameters yielding R^2^X = 0.942, R^2^Y = 0.976, and Q^2^ = 0.962. These metrics indicated that the PLS-DA model possessed substantial explanatory power and robust predictive capability for distinguishing ham samples among different treatment groups, effectively capturing the intergroup variability in flavor compound profiles. The validation and over-fitting of the PLS-DA model were evaluated by a permutation test (*n* = 200, Fig. 2c). The results demonstrated that the Q^2^ values of all random permutation models were significantly lower than the original model, and the Q^2^ regression line was less than zero in the longitudinal axis intercept. At the same time, the Q^2^ value decreases with the decrease in permutation retention. The results indicated that the original model had good stability, a characteristic that prevented over-fitting in the analysis, and reliable prediction ability, which could effectively reveal the different characteristics of volatile flavor compounds in hams of different treatment groups.

#### 3.4.3 Screening of volatile characteristic flavor compounds

Within the PLS-DA framework, the Variable Importance in the Projection (VIP) metric quantifies the contribution of each variable to the classification of sample groups. Variables with VIP ≥ 1 are typically recognized as exerting a critical influence on the discriminant process (Li, Zou, et al., 2024; Zhang et al., 2019). Under these conditions, a total of 32 potential volatile characterization markers were screened in this study (Table 2), including 9 alcohols, 9 aldehydes, 5 esters, 5 ketones, 2 acids, and 2 terpenes, which aligns with our previous findings (Li, Zou, et al., 2024). To visually characterize the discrepancies in flavor markers among different ham groups, a correlation heat map analysis was conducted (Fig. 2d). The results revealed that flavor markers of the M-US and H-US groups clustered into one distinct category, while those of the CG, LO, and L-US groups formed a separate clustering category. This clustering pattern indicates that combined moderate- and high-intensity ultrasound treatment with LO (M-US, H-US) induces convergent changes in the composition and abundance of flavor markers. This suggests that moderate- and high-intensity ultrasound may drive significant flavor profile reshaping associated with aldehyde metabolic dynamics.

**Table 2:** Screening of characteristic flavor marker substances based on peak intensity of Nuodeng ham.

Compounds	VIP value	Peak intensity value/mV				
		CG	LO	L-US	M-US	H-US
Heptanal	1.37	192.64 ± 16.75^c^	246.24 ± 17.91^b^	192.07 ± 9.34^c^	271.60 ± 17.16^a^	230.23 ± 14.70^b^
4-Methyl-3-penten-2-one	1.32	41.75 ± 2.22^c^	33.98 ± 5.39^cd^	32.55 ± 2.99^d^	275.77 ± 11.76^a^	65.95 ± 8.26^b^
Hexanal-M	1.28	1346.10 ± 85.12^c^	1598.66 ± 29.62^a^	1246.76 ± 33.89^d^	1421.35 ± 54.93^b^	1479.09 ± 35.50^b^
Hexanal-D	1.27	770.92 ± 83.85^c^	1851.62 ± 215.51^a^	623.50 ± 71.18^d^	860.95 ± 73.94^c^	1012.79 ± 69.36^b^
Acetoin-D	1.26	1063.11 ± 84.75^c^	1142.62 ± 106.45^c^	1301.92 ± 93.37^b^	1322.19 ± 95.80^b^	1473.51 ± 75.00^a^
1-Penten-3-ol	1.23	2384.41 ± 67.95^b^	2266.02 ± 50.90^c^	1875.79 ± 54.91^e^	2036.54 ± 21.58 ^d^	2826.69 ± 30.65^a^
3-Methyl-2-butanol	1.22	76.38 ± 7.12^b^	39.59 ± 2.53^e^	46.40 ± 2.61^d^	95.69 ± 6.24^a^	57.56 ± 4.55^c^
2-Pentanone	1.21	2173.06 ± 104.53^c^	2205.20 ± 83.84^c^	2068.37 ± 89.05^d^	2803.53 ± 65.19^b^	3286.38 ± 62.02^a^
(E)-2-Pentenal	1.20	149.57 ± 6.43^c^	174.62 ± 4.80^b^	135.78 ± 8.65^d^	146.50 ± 7.87^cd^	200.01 ± 17.29^a^
Ethyl Acetate	1.20	10,666.56 ± 193.97^c^	11,124.42 ± 110.98^b^	10,731.33 ± 182.01^c^	11,026.12 ± 218.84^b^	11,747.21 ± 145.59^a^
1-Hexanol	1.20	694.18 ± 59.83^c^	1581.66 ± 204.78^a^	758.17 ± 35.62^c^	1155.135 ± 31.41^b^	1170.29 ± 64.34^b^
2-Methyl-1-propanol-D	1.20	1212.25 ± 53.85^b^	1356.58 ± 36.34^a^	1205.52 ± 26.79^b^	1343.78 ± 15.18^a^	1314.15 ± 40.77^a^
3-Methyl-1-butanol-M	1.18	3287.05 ± 140.84^b^	3546.29 ± 101.56^a^	3052.73 ± 30.08^c^	3097.45 ± 74.96^c^	3278.75 ± 56.81^b^
Propyl acetate	1.18	247.00 ± 10.01^b^	305.51 ± 16.54^a^	234.98 ± 16.38^b^	231.58 ± 14.88^b^	241.50 ± 5.48^b^
Pentanol	1.17	565.60 ± 75.27^c^	727.98 ± 67.30^b^	465.13 ± 34.06^d^	598.47 ± 15.00^c^	789.97 ± 36.50^a^
3-Methyl-1-butanol-D	1.17	1910.49 ± 166.67^b^	2200.26 ± 184.53^a^	1588.63 ± 52.76^c^	1678.20 ± 43.04^c^	1905.38 ± 87.89^b^
tert-Butanol	1.16	1196.22 ± 32.39^b^	1356.74 ± 25.36^a^	1215.07 ± 23.90^b^	1179.19 ± 47.27^b^	1361.08 ± 29.32^a^
Acetic acid-M	1.14	9908.24 ± 353.13^b^	11,062.46 ± 432.02^a^	11,307.97 ± 201.41^a^	10,154.44 ± 362.37^b^	10,954.76 ± 217.42^a^
2-Heptanone	1.12	453.48 ± 26.33^d^	852.11 ± 106.79^a^	521.18 ± 28.49^c^	595.06 ± 31.79^b^	596.39 ± 13.30.13^b^
beta-Pinene	1.11	450.89 ± 48.45^d^	982.49 ± 109.57^c^	1065.39 ± 80.40^c^	2461.33 ± 33.97^a^	1242.98 ± 81.48^b^
2-Butanone	1.11	4017.90 ± 240.46^d^	6357.45 ± 72.26^a^	4964.74 ± 105.29^c^	5527.27 ± 136.07^b^	5576.63 ± 169.58^b^
(E)-2-Hexenal	1.10	96.43 ± 5.25^c^	60.53 ± 4.06^d^	56.83 ± 1.12^d^	133.95 ± 8.70^a^	117.94 ± 6.80^b^
Ethyl pentanoate	1.09	215.36 ± 9.48^b^	193.19 ± 9.18^c^	202.62 ± 8.92^c^	254.92 ± 7.04^a^	264.73 ± 14.64^a^
Pentanal	1.08	168.39 ± 12.61^b^	200.59 ± 7.91^a^	146.62 ± 7.48^c^	115.61 ± 4.55^d^	156.65 ± 8.68^c^
2-Methylpropyl acetate	1.07	2218.39 ± 27.10^c^	2410.36 ± 44.36^b^	2590.90 ± 21.83^a^	2570.64 ± 46.98^a^	2550.92 ± 46.50^a^
Isobutanoic acid	1.06	1025.38 ± 106.45^bc^	1001.63 ± 80.64^bc^	923.73 ± 89.32^c^	1042 ± 76.59^b^	1234.01 ± 42.87^a^
Propanal-D	1.06	1690.84 ± 73.60^a^	1417.13 ± 59.21^b^	1001.36 ± 57.25^d^	1018.88 ± 45.87^d^	1162.75 ± 98.93^c^
Ethyl hexanoate	1.05	443.22 ± 42.23^d^	395.83 ± 40.29^d^	545.21 ± 78.18^c^	702.82 ± 29.34^a^	612.79 ± 26.13^b^
Propanal-M	1.04	1756.12 ± 61.90^a^	1623.77 ± 27.68^ab^	1600.76 ± 36.89^b^	1335.89 ± 233.07^c^	1228.45 ± 43.16^c^
Acetic acid-D	1.04	2911.97 ± 307.26^c^	4211.34 ± 428.38^a^	4383.78 ± 299.36^a^	3182.83 ± 320.35^c^	3824.44 ± 178.52^b^
Camphene	1.03	91.22 ± 12.81^d^	184.45 ± 16.99^c^	171.07 ± 9.66^c^	299.52 ± 8.03^a^	203.82 ± 9.04^b^
2-Methyl-1-propanol-M	1.02	1640.93 ± 47.60^c^	1483.64 ± 18.7^e^	1561.56 ± 16.38^d^	1749.23 ± 37.89^a^	1711.51 ± 14.88^b^

Note: In the same row, data with different lowercase letters indicate significant differences (*P* < 0.05).

As characteristic degradation products of lipid oxidation, aldehydes are key volatile flavor compounds in ham (Li et al., 2022). Hexanal has a relatively low threshold in meat products, has a grassy flavor, and contributes significantly to ham flavor formation. However, excessive amounts of hexanal can produce a rancid odor due to excessive fat oxidation, which can have a negative effect on product flavor (Zhou et al., 2020; Zhou et al., 2025). In this work, the LO group exhibited a relatively high hexanal content, and its content showed a downward trend after ultrasonic treatment. In the study of ultrasonic treatment of spiced beef, Zou et al. (2018) found that when the test power increased from 600 W to 800 W, the hexanal content decreased significantly, which was similar to the results of this study. It is noteworthy that the contents of pentanal and propanal-M were significantly higher in the LO and CG groups. With the increase of ultrasonic power, the content of these substances in the L-US and M-US treatment groups showed a downward trend, while the content in the H-US group showed an increasing trend. This may result from the fact that sustained ultrasound treatment induces denaturation of myofibrillar proteins, which in turn exposes more hydrophobic groups for covalent binding reactions with aldehydes, leading to a decrease in free aldehyde content; whereas, when sustained high-power ultrasound energy exceeds the critical threshold, complete depolymerization of the protein network structure occurs, releasing bound aldehydes, while facilitating the secondary generation of lipid oxidation end-products, such as aldehydes (Zhang, Xing, et al., 2021; Zhang, Zhang, & Xing, 2021).

Ketones, as the products of further oxidation of aldehydes or the products of the Melad reaction, have a slightly higher olfactory threshold than aldehydes and can present a creamy aroma and fresh fruity flavor. Among them, 2-ketones play a key role in the flavor formation of dry-cured ham (Li, Zou, et al., 2024). In this study, 2-heptanone, 2-butanone, and 2-pentanone were identified as differentiated characteristic flavor substances among the groups. The contents of all the above substances in the LO group were markedly higher than those in the CG group, and their contents showed a trend of first decreasing and then increasing with increasing ultrasonic power. The cause of this phenomenon can be a decrease due to accelerated oxidation at moderate ultrasound power, but an increase at higher power due to enhanced lipid oxidation and protein degradation. This is in agreement with the findings reported by Zou et al. (2018).

Alcohols are mostly originated from fat oxidation or microbial metabolism, with high threshold, often with herbal and woody aroma, and can be used as flavor precursors or auxiliary components; esters are generated by esterification of fatty acids and alcohols, with low threshold, and presenting fruity and floral aroma, which are the key contributors to the characteristic flavor of meat (Liu et al., 2020; Shi et al., 2019). Among the characteristic flavor substances screened in this study, the total content of alcohols and esters increased significantly after ultrasonic treatment. The mechanical shear force and cavitation bubble burst energy generated by ultrasound can accelerate the oxidation of fat to alcohol precursors and promote the esterification reaction of fatty acids and alcohols. The antioxidant components of the compound essential oil of *Litsea pungens Hemsl* may indirectly maintain the accumulation of alcohols and esters by inhibiting lipid overoxidation or regulating microbial metabolism, which had a positive effect on the flavor and aroma formation of ham (Bao et al., 2023; Zou et al., 2018).

### 3.5 Analysis of small molecule metabolites

The metabolites of ham samples from different treatment groups were analyzed by a UPLC-QE-MS non-targeted metabolomics method, aiming to investigate the metabolic differences between groups. 249 (ESI+) and 325 (ESI−) metabolites were identified from the ham samples in each group, including amino acids, peptides and analogs, fatty acids, nucleosides, sugars, vitamins, etc.

#### 3.5.1 PCA and PLS-DA analysis

**Fig. 3:**
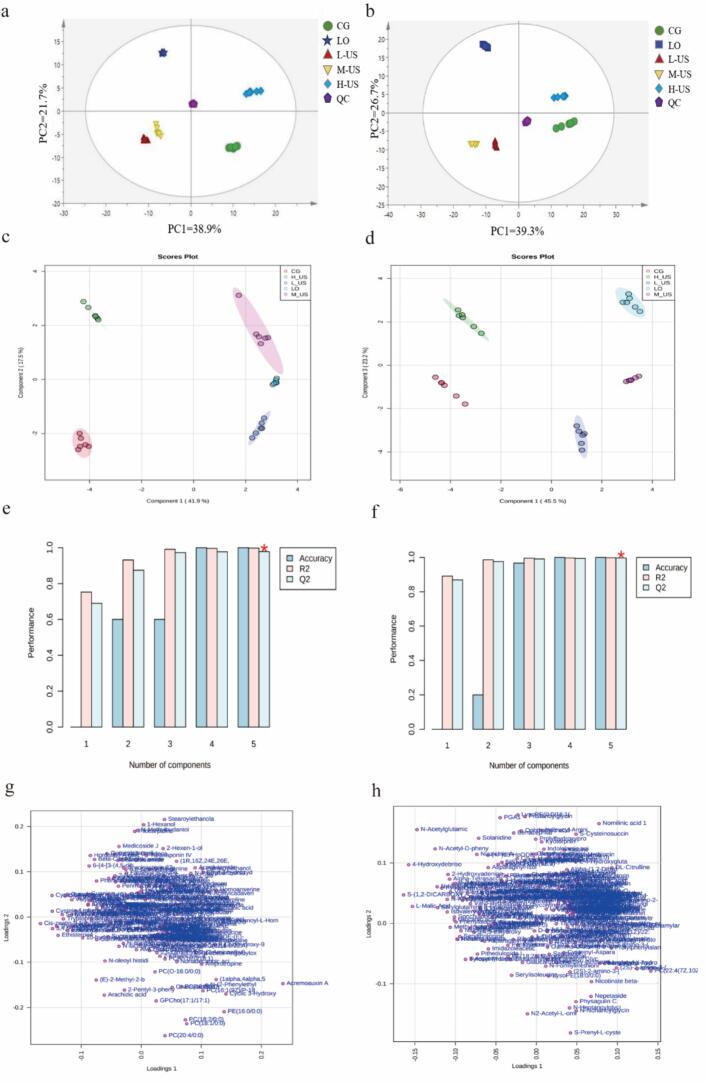
Analysis of small molecule metabolites in the Nuodeng ham by UHPLC-QE- LC/MS. PCA score plots and OPLS-DA score plots of all experimental samples in ESI+ (a, c) and ESI− mode (b, d), and permutations plots of PLS-DA models of ESI+ (e) and ESI− (f) mode, loading plot of two groups in ESI+ (g) and ESI− mode (h).

PCA, as a multivariate statistical method, characterizes complex variables with principal component factors by downscaling high-dimensional data, and then assesses the differences between different samples based on principal component contributions (Li et al., 2019). As depicted in Fig. 2, all samples were within the 95 % confidence interval. The PCA model exhibited cumulative variance explained values of 60.6 % (ESI+, Fig. 3a) and 66 % (ESI−, Fig. 3b), revealing a distinct separation trend among sample groups and indicating divergent metabolic profiles across different treatment groups of ham samples.

PLS-DA revealed significant metabolic differences between treatment groups, with 59.4 % (ESI+, Fig. 3c) and 66 % (Fig. 3d) variance explained. Score plots showed clear intergroup separation, confirming distinct metabolic profiles across treatments. Cross-validation analysis demonstrated robust model performance in both ionization modes: ESI+ mode exhibited R^2^X = 0.998 and Q^2^ = 0.979 (Fig. 3e), while ESI− mode achieved R^2^X = 0.998 and Q^2^ = 0.997 (Fig. 3f). These metrics confirm the model's strong cumulative explanatory power and exceptional predictive capacity, indicating reliable discrimination of metabolic variations. As depicted in Fig. 3g-h, the load diagram exhibited pronounced intergroup variations, where metabolites positioned further from the centroid substantially impacted sample compositional disparities (Zhou et al., 2025). In this study, compounds such as acremoauxin A, cysteinyl-Leucine, isoleucylproline, arginine, etc. emerged as key contributors to sample discrimination in ESI+ mode (Fig. 3g), while compounds such as 4-hydroxydebrisoquine, L-malic acid, N-(alpha-Linolenoyl), tyrosine, etc. had significant contributions to sample classification in ESI− mode (Fig. 3h). These metabolites may be hypothesized to serve as critical biochemical markers driving the observed differences between treatment groups.

#### 3.5.2 Screening and analysis of differential metabolites

**Fig. 4:**
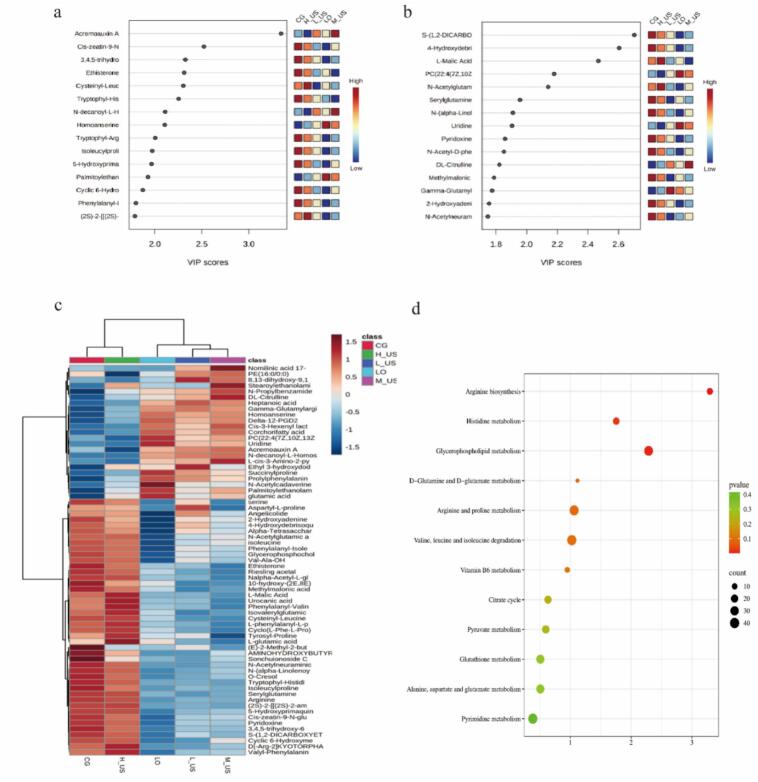
Differential metabolites in the Nuodeng ham. VIP score diagram in ESI+ (a) and ESI− mode (b); (c) Cluster heatmap of differential small molecule compounds in different treatment groups; (d) Pathway enrichment analysis of differential metabolites;

Metabolites with a VIP value greater than 1 were identified as key differential metabolites, which were crucial in distinguishing metabolic differences among hams from different treatment groups (Zhou et al., 2025). A total of 33 and 34 differential metabolites were identified in positive and negative ion modes, respectively, using VIP scores from the PLS-DA model with significance criteria of VIP ≥ 1.5 and *P* < 0.05. As depicted in Fig. 4a, the VIP values of acremoauxin A, cysteinyl-leucine, tryptophyl-histidine, N-decanoyl-L-Homoserine lactone, and homoanserine significantly exceeded 1 under ESI+ mode. And metabolites such as S-(1,2-DICARBOXYETHYL) GLUTATHIONE, 4-hydroxydebrisoquine, L-malic acid, and *N*-acetylglutamic acid exhibited VIP values notably greater than 1 in the ESI− mode (Fig. 4b). These findings were consistent with the results presented in the aforementioned load diagrams, reinforcing the discriminatory power of these metabolites across different ionization modes.

A total of 67 differential metabolites were screened under the two modes, including amino acids and peptides and analogues, fatty acids, nucleosides, sugars, organic acids, etc. (Table S4). In the long processing cycle of dry-cured hams, proteins are extensively hydrolyzed under the action of enzymes to produce characteristic flavor precursors with free amino acids as the core, supplemented by active components such as small peptides, which together constitute the metabolic basis for the formation of their unique taste (Feng et al., 2018). Research has demonstrated that amino acids serve as essential precursors in the formation of ham flavor, directly influencing its overall flavor profile (Lin et al., 2020). In this work, arginine, isoleucine and other bitter amino acids in ham were the highest in the CG group. With ultrasonic treatment, the content of these bitter amino acids gradually decreased. Secondly, the content of unami amino acids such as L-glutamic acid and glutamic acid was the highest in the LO group. This may be because the composite coating may selectively inhibit the activity of glutaminase in the proteolytic enzyme system by releasing essential oil components and scavenging free radicals, reducing the conversion of glutamic acid to other metabolites, and maintaining protease activity to continuously release free glutamic acid (Hu et al., 2019). In addition, the contents of N-decanoyl-L-homoserine lactone, palmitoylethanolamide (PEA), and uridine were higher in LO, L-US, and M-US groups. Among them, N-decanoyl-L-homoserine lactone belongs to the acyl homoserine lactone (AHLs) family. It plays a key role in flavor formation, microbial safety and texture improvement by coordinating bacterial metabolic activities in ham (Xiong et al., 2020). PEA is an endogenous fatty acyl ethanolamine, which is widely found in animal tissues and some foods. It has weak antioxidant activity and can inhibit lipid peroxidation by scavenging free radicals or chelating metal ions, which may delay the oxidation of unsaturated fatty acids in ham and reduce the formation of odorous substances. At the same time, it avoids the hardening of texture caused by oxidation and maintains the structure of muscle protein, which is beneficial to the improvement of ham texture (Gallelli et al., 2018). Studies have shown that uridine, as a precursor of umami substances, can produce nucleotide flavor substances by degradation or metabolism, and produce a synergistic effect with free amino acids in meat to enhance the umami intensity and richness of meat products (Khan et al., 2015). Additionally, uridine can inhibit the activity of fat oxidase, slow down the unpleasant flavor produced by the oxidation of meat fat, and at the same time, through chelating metal ions, slow down the reaction of lipid peroxidation and maintain the original flavor of meat products (Lee et al., 2020). These results indicated that medium-power ultrasound combined with the *Litsea pungens Hemsl* essential oil compound coatings treatment can significantly affect the composition and content of ham metabolites, promote the production of beneficial flavor substances and inhibit the accumulation of lipid oxidation-related metabolites.

Based on the peak area characterization of the metabolite contents, a hierarchical cluster analysis was performed to identify the significant differential metabolites in the ham samples (Fig. 4c). The results showed that the levels of different metabolites exhibited specific distributions across groups, and the cluster analysis showed that the CG group and the H-US group were clustered into one group. In contrast, the LO, L-US, and M-US groups formed another, with both ultimately merging into a single large cluster. Methylmalonic acid, L-malic acid, urocanic acid, and phenylalanyl-valine were higher in the CG and H-US groups. At the same time, acremoauxin A, N-decanoyl-L-homoserine lactone, palmitoylethanolamide, and uridine were higher in LO, L-US, and M-US. These findings highlight that whereas LO and L-US treatments improved certain flavor aspects, the medium-power ultrasound combined with LO (M-US) provided a more balanced flavor profile by simultaneously enhancing desirable compounds and reducing undesirable bitter and oxidation-related metabolites.

#### 3.5.3 Metabolic pathway analysis

To explore the potential metabolic pathways of differential metabolites in the treatment groups with different power ultrasounds combined with *Litsea pungens Hemsl* essential oil compound coatings, KEGG enrichment analysis of key metabolites was carried out. A sum of 20 different metabolic pathways was obtained through enrichment analysis. As depicted in Fig. 4d, under the screening condition of impact value >0.01, 12 key metabolic pathways were identified, including glycerophospholipid metabolism, arginine biosynthesis, pyruvate metabolism, glutathione metabolism, pyrimidine metabolism, etc., and 10 metabolites were accurately mapped, namely L-glutamic acid, L-arginine, DL-citrulline, uric acid, phosphatidylcholine, L-malic acid, glycerophosphocholine, methylmalonic acid, pyridoxine, and uridine. Notably, L-glutamate was involved in 11 metabolic pathways, while phosphatidylcholine participated in 4 pathways. Both L-arginine and L-malic acid were implicated in 3 pathways, respectively. It can be concluded that these differential metabolites resided in the intersection of multiple metabolic pathways, enabling them to exert a greater influence on ham's metabolic pathways and quality.

**Fig. 5:**
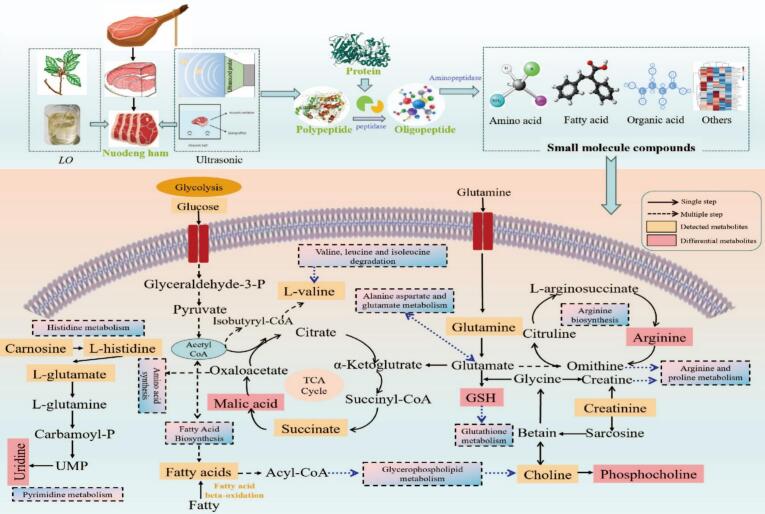
Analysis of the effect of ultrasound combined with *Litsea pungens Hemsl* essential oil compound coating on small molecule metabolic pathways network, yellow represents the metabolites identified in this study, and red represents the key metabolites identified. (For interpretation of the references to color in this figure legend, the reader is referred to the web version of this article.)

Based on the annotation and analysis of metabolic pathways in the KEGG database, the regulation mechanism of ultrasound combined with essential oil coating treatment on the metabolic network of small molecular metabolites in Nuodeng ham was further clarified. As shown in Fig. 5, ultrasonic combined with LpH essential oil coating treatment can effectively regulate the late metabolic process of Nuodeng ham. This treatment significantly activated the amino acid metabolic pathways closely related to flavor formation, especially the metabolism of glutamic acid, aspartic acid, and branched-chain amino acids. At the same time, changes in the quality of ham will affect the production of fatty acids, prompting more fatty acids to be converted into acetyl-CoA by β-oxidation and enter the TCA cycle, thereby affecting the dynamics of related metabolites (Zhou et al., 2025). In addition, free fatty acids produced by phospholipid degradation can be further oxidized to volatile flavor substances such as aldehydes and ketones, indicating that changes in glycerophospholipid metabolism may change the degree of lipid oxidation or the formation of specific flavor lipid precursors, thereby affecting the formation of ham flavor (Zheng et al., 2024). Additionally, activating glutathione metabolism combats oxidative damage, delaying lipid peroxidation and protein oxidation, and this activation forms the key metabolic basis for maintaining ham's color, flavor, and nutritional quality. The synergistic changes of these metabolic networks jointly promoted the formation and accumulation of characteristic flavor substances of Nuodeng ham, inhibited the adverse oxidation reaction, and finally achieved the effect of improving the overall flavor quality of ham and enhancing the stability of the product.

## 4 Conclusion

This study systematically evaluated the effects of different ultrasound power levels combined with *Litsea pungens Hemsl* essential oil (LO) composite coating on Nuodeng ham quality. Results showed that compared with the control group, both the LO group and ultrasound-LO combined groups significantly increased ham a* value and decreased b* value, improving color characteristics. The H-US group exhibited notably reduced hardness, attributed to ultrasound cavitation disrupting muscle tissue; notably, medium-power ultrasound (200–400 W) combined with LO achieved a better flavor-texture balance. GC-IMS analysis indicated that low and medium power ultrasound promoted the formation of characteristic flavor substances, with the M-US group showing balanced aldehyde and ester contents. In contrast, high-power ultrasound caused aldehyde-protein binding or secondary oxidation, altering flavor profiles. Metabolomics results further revealed that the combined treatment regulated amino acid metabolism, TCA cycle, and lipid metabolism pathways, promoting umami amino acids, bacteriostatic N-decanoyl-L-homoserine lactone, and antioxidant palmitoylethanolamide while inhibiting bitter amino acid production. In conclusion, 400 W ultrasound combined with LO yielded the optimal overall Nuodeng ham quality, representing the best parameter for balancing color, texture, and flavor.

## CRediT authorship contribution statement

**Nannan Zhou:** Writing – review & editing, Writing – original draft, Software, Methodology, Data curation, Conceptualization. **Lihong Zhang:** Methodology, Investigation, Formal analysis, Data curation. **Guiying Wang:** Validation, Resources, Project administration, Methodology. **Ruwei Ren:** Methodology, Conceptualization. **Yanfei Du:** Software, Methodology. **Shuai Tang:** Visualization, Conceptualization. **Jiayan Tan:** Methodology, Investigation. **Guozhou Liao:** Validation, Resources, Project administration, Funding acquisition.

## Declaration of competing interest

The authors declare that they have no known competing financial interests or personal relationships that could have appeared to influence the work reported in this paper.

## Data Availability

The authors do not have permission to share data.

## Appendix A Supplementary data

Supplementary material Image 1
