# Supplementary material for: Exploring ultrasound combined with Litsea pungens Hemsl essential oil compound coating for modulating flavor and metabolites in Nuodeng ham: A multidimensional analysis by UHPLC-QE-MS and GC-IMS
Source: Food Chem X. 2025 Dec 11;33:103404. doi: 10.1016/j.fochx.2025.103404 (PMC12774757; doi:10.1016/j.fochx.2025.103404)
Supplement: Supplementary file 1 — Supplementary material [file mmc1.docx]

Table S1 GC-IMS analysis parameter condition

| Items | | Parameters |
| --- | --- | --- |
| Autosampler conditions | Headspace incubation temperature | 60 ℃ |
|  | Incubation time | 20 min |
|  | Heating method | Oscillating heating |
|  | Needle temperature | 85 ℃ |
|  | Injection volume | 500 μL |
|  | Incubation speed | 500 rpm |
| GC conditions | Column type | MXT-WAX, ID: 0.53 mm |
|  | Column temperature | 60 ℃ |
|  | Carrier gas | N_2_，Purity≥99.999% |
|  | Running time | 30 min |
|  | Carrier gas flow rate | 0-2 min,，2 mL/min  2-10 min，2 mL/min-20 mL/min  10-20 min，20 mL/min-100 mL/min  20-30 min，100 mL/min |
| IMS conditions | Ionisation source  Radioactive intensity  In-tube linear voltage  IMS temperature | β-ray (tritium ^3^H)  300 MBq  5000 V  45 ℃ |
|  | Drift gas  Drift gas flow | N_2_，Purity≥99.999%  150 mL/min |
|  | Drift tube length | 98 mm |

Table S2 Chromatographic and mass spectrometric conditions

|  | Items | Parameters |
| --- | --- | --- |
| Chromatographic conditions | Chromatographic column | ACQUITY UPLC HSS T3 |
|  | Mobile phase A | 95% water + 5% acetonitrile (containing 0.1% formic acid) |
|  | Mobile phase B | 47.5% acetonitrile + 47.5% isopropyl alcohol + 5% water (containing 0.1% formic acid) |
|  | Injection volume | 2 μL |
|  | Column temperature | 40 ℃ |
| Mass spectrometric conditions | Scanned area | 70-1050 (m/z) |
|  | Sheath gas flow rate | 50 (arb) |
|  | Auxiliary gas flow rate | 13 (arb) |
|  | Ion source heating temperature | 450 ℃ |
|  | Ionization voltage (positive electrode) | 3500 (V) |
|  | Ionization voltage (negative electrode) | -3500 (V) |
|  | Capillary temperature | 325 ℃ |
|  | S-Lens voltage | 50 V |
|  | Resolution (Full MS) | 60000 |
|  | Resolution (MS2) | 7500 |
|  | Collision energy | 20,40,60 (eV) |

Table S3 Qualitative analysis of volatile components in Nuodeng ham

| Type | Number | Compounds | | CAS | Retention index | Retention time/s | Migration time/ms |
| --- | --- | --- | --- | --- | --- | --- | --- |
|  | 7 | 1-Hexanol | | C111273 | 1369.4 | 957.28 | 1.32992 |
|  | 11 | Pentanol | | C5H12O | 264.8 | 765.26 | 1.25826 |
|  | 13 | 3-Methyl-1-butanol-M | | C123513 | 1222.1 | 700.45 | 1.24552 |
|  | 14 | 3-Methyl-1-butanol-D | | C123513 | 1222.1 | 700.45 | 1.48855 |
|  | 15 | | 1-Penten-3-ol | C616251 | 1176.7 | 625.52 | 0.94135 |
|  | 17 | | 1-Butanol-M | C71364 | 1162.1 | 595.98 | 1.18289 |
| Alcohols | 18 | | 1-Butanol-D | C71363 | 1161.6 | 594.92 | 1.37821 |
|  | 22 | | 2-Methyl-1-propanol-M | C78831 | 1108.4 | 498.73 | 1.17115 |
|  | 23 | | 2-Methyl-1-propanol-D | C78831 | 1107.9 | 497.92 | 1.36579 |
|  | 36 | | 2-Butanol | C78922 | 1027.4 | 390.69 | 1.33833 |
|  | 38 | | Ethanol | C64175 | 944.3 | 316.75 | 1.13409 |
|  | 47 | | 3-Methyl-2-butanol | C598754 | 1121.8 | 521.381 | 1.23395 |
|  | 49 | | tert-Butanol | C75650 | 923.5 | 302.314 | 1.32533 |
|  | 54 | | 1,8-Cineole | C470826 | 1210.6 | 683.929 | 1.30205 |
|  | 60 | | 1-Propanol | C71238 | 1052.7 | 421.134 | 1.25234 |
|  | 6 | | (E)-2-Octenal | C2548870 | 1412.5 | 1051.13 | 1.34825 |
|  | 19 | | (E)-2-Hexenal | C6728263 | 1211.1 | 684.62 | 1.20228 |
|  | 20 | | Heptanal | C111717 | 1196.6 | 664.42 | 1.33356 |
|  | 24 | | Hexanal-M | C66251 | 1099 | 483.30 | 1.26653 |
| Aldehydes | 25 | | Hexanal-D | C66251 | 1100 | 484.92 | 1.563 |
|  | 37 | | 3-Methylbutanal | C590863 | 927.6 | 305.08 | 1.40283 |
|  | 42 | | Propanal-M | C123386 | 814.3 | 236.333 | 1.05645 |
|  | 43 | | Propanal-D | C123386 | 817.9 | 238.279 | 1.14723 |
|  | 46 | | (E)-2-Pentenal | C1576870 | 1115.1 | 509.961 | 1.09217 |
|  | 48 | | 2-Methylpropanal | C78842 | 821.4 | 240.179 | 1.2802 |
|  | 61 | | Pentanal | C110623 | 992.9 | 353.432 | 1.42587 |
|  | 62 | | Butanal | C123728 | 886.4 | 278.009 | 1.27857 |
|  | 1 | | Propanoic acid | C79094 | 1638.6 | 1717.00 | 1.11155 |
|  | 2 | | Isobutanoic acid | C79312 | 1628.6 | 1680.27 | 1.14967 |
| Acids | 4 | | Acetic acid-M | C64197 | 1503.6 | 1280.89 | 1.05865 |
|  | 5 | | Acetic acid-D | C64197 | 1503 | 1279.30 | 1.16069 |
|  | 9 | | Acetoin-M | C513860 | 1300.1 | 823.65 | 1.06034 |
|  | 10 | | Acetoin-D | C513860 | 1299.4 | 822.35 | 1.33162 |
|  | 16 | | 2-Heptanone | C110430 | 1193.6 | 660.35 | 1.26192 |
|  | 29 | | 1-Penten-3-one | C1629589 | 1069 | 441.88 | 1.08479 |
|  | 31 | | 4-Methyl-2-pentanone | C108101 | 1024.6 | 387.44 | 1.49003 |
|  | 32 | | 2-Pentanone | C107879 | 999.4 | 359.56 | 1.36939 |
|  | 39 | | 2-Butanone | C78933 | 914.2 | 296.00 | 1.24517 |
| Ketones | 41 | | Acetone | C67641 | 838 | 249.30 | 1.11378 |
|  | 51 | | 6-Methyl-5-hepten-2-one | C110930 | 1347 | 911.877 | 1.17775 |
|  | 52 | | 2-Octanone | C111137 | 1345.2 | 908.436 | 1.32331 |
|  | 58 | | 4-Methyl-3-penten-2-one | C141797 | 1134.6 | 544.037 | 1.44454 |
|  | 59 | | 1-Octen-3-one | C4312996 | 1308.2 | 838.345 | 1.27566 |
|  | 3 | | Ethyl 3-hydroxybutanoate | C5405414 | 1563.1 | 1457.40 | 1.17111 |
|  | 8 | | Ethyl lactate | C97643 | 1359.3 | 936.52 | 1.14906 |
|  | 12 | | Ethyl hexanoate | C8H16O2 | 1243.4 | 732.11 | 1.34243 |
|  | 26 | | Ethyl3-methylbutanoate-M | C108645 | 1079.3 | 455.68 | 1.26524 |
|  | 27 | | Ethyl 3-methylbutanoate-D | C108645 | 1080.5 | 457.31 | 1.6571 |
|  | 28 | | Ethyl 2-methylbutanoate | C7452791 | 1066.5 | 438.63 | 1.64807 |
| Esters | 30 | | Ethyl butanoate | C105544 | 1053.3 | 421.82 | 1.5593 |
|  | 33 | | 2-Methylpropyl acetate | C110190 | 998.2 | 358.26 | 1.22845 |
|  | 34 | | Ethyl isobutyrate | C97621 | 981.7 | 344.64 | 1.56169 |
|  | 35 | | Ethyl propanoate | C105373 | 969.9 | 335.56 | 1.453 |
|  | 40 | | Ethyl Acetate | C141786 | 896.3 | 284.33 | 1.33595 |
|  | 45 | | Ethyl formate | C109944 | 824.1 | 241.629 | 1.20842 |
|  | 50 | | Propyl acetate | C109604 | 992.2 | 352.878 | 1.46976 |
|  | 56 | | Ethyl pentanoate | C539822 | 1149.9 | 572.308 | 1.27498 |
|  | 21 | | beta-Pinene | C127913 | 1132 | 539.34 | 1.21497 |
| Terpenes | 55 | | alpha-Phellandrene | C99832 | 1175.3 | 622.616 | 1.21748 |
|  | 57 | | Camphene | C79925 | 1115.7 | 510.876 | 1.21653 |
| Pyrazine | 53 | | 2,5-Dimethylpyrazine | C123320 | 1313.9 | 848.792 | 1.11121 |
| Thioether | 44 | | Dimethyl sulfide | C75183 | 759.2 | 208.754 | 0.95913 |

Table S4 Differential metabolites between different treatment groups

| Mode | metabolites | RT/s | m/z | VIP | *P* value |
| --- | --- | --- | --- | --- | --- |
| ESI+ | Acremoauxin A [(2R,3R,4R) | 306.137 | 5.77 | 3.34 | <0.001 |
|  | Cis-zeatin-9-N-glucoside | 423.197 | 2.54 | 2.52 | <0.001 |
|  | 3,4,5-trihydroxy-6-{[(3E)-4-phenylbut-3-en-2- | 342.156 | 2.16 | 2.33 | <0.001 |
|  | Ethisterone | 330.246 | 6.94 | 2.31 | <0.001 |
|  | Cysteinyl-Leucine | 276.137 | 1.15 | 2.30 | <0.001 |
|  | Tryptophyl-Histidine | 324.146 | 3.04 | 2.25 | <0.001 |
|  | N-decanoyl-L-Homoserine lactone | 256.190 | 6.26 | 2.11 | <0.001 |
|  | Homoanserine | 318.154 | 4.80 | 2.11 | <0.001 |
|  | Arginine | 140.082 | 2.12 | 2.00 | <0.001 |
|  | Isoleucylproline | 229.155 | 2.85 | 1.97 | <0.001 |
|  | 5-Hydroxyprimaquine | 276.170 | 2.79 | 1.97 | <0.001 |
|  | Palmitoylethanolamide | 300.289 | 6.90 | 1.93 | <0.001 |
|  | Cyclic 6-Hydroxymelatonin | 515.192 | 2.19 | 1.87 | <0.001 |
|  | Phenylalanyl-Isoleucine | 261.159 | 6.18 | 1.80 | <0.001 |
|  | (2S)-2-[[(2S)-2-aminopropanoyl]amino]-4 | 203.139 | 2.73 | 1.79 | <0.001 |
|  | D[-Arg-2]KYOTORPHAN | 338.182 | 1.21 | 1.75 | <0.001 |
|  | Glycerophosphocholine | 280.092 | 0.64 | 1.74 | <0.001 |
|  | PE(16:0/0:0) | 454.293 | 6.79 | 1.73 | <0.001 |
|  | Heptanoic acid | 194.115 | 4.63 | 1.73 | <0.001 |
|  | L-phenylalanyl-L-proline | 263.139 | 3.97 | 1.73 | <0.001 |
|  | glutamic acid | 189.087 | 0.85 | 1.70 | <0.001 |
|  | Stearoylethanolamide | 328.321 | 7.05 | 1.70 | <0.001 |
|  | 8,13-dihydroxy-9,11-octadecadienoic acid | 313.237 | 6.43 | 1.70 | <0.001 |
|  | (E)-2-Methyl-2-buten-1-ol  O-beta-D-Glucopyranoside | 538.287 | 3.61 | 1.67 | <0.001 |
|  | 10-hydroxy-(2E,8E)-decadien-4-ynoic Acid | 222.112 | 5.61 | 1.67 | <0.001 |
|  | AMINOHYDROXYBUTYRIC ACID | 120.066 | 0.68 | 1.67 | <0.001 |
|  | N-Propylbenzamide | 181.133 | 6.23 | 1.64 | <0.001 |
|  | Valyl-Phenylalanine | 287.136 | 3.66 | 1.62 | <0.001 |
|  | Ethyl 3-hydroxydodecanoate | 262.237 | 5.86 | 1.61 | <0.001 |
|  | N-Acetylcadaverine | 145.134 | 1.15 | 1.61 | <0.001 |
|  | Succinylproline | 216.087 | 2.89 | 1.58 | <0.001 |
|  | Cyclo(L-Phe-L-Pro) | 245.128 | 4.98 | 1.56 | <0.001 |
|  | Cis-3-Hexenyl lactate | 190.144 | 3.87 | 1.54 | <0.001 |
| ESI- | S-(1,2-DICARBOXYETHYL)Glutathione | 433.136 | 3.06 | 2.70 | <0.001 |
|  | 4-Hydroxydebrisoquine | 500.919 | 0.54 | 2.61 | <0.001 |
|  | L-Malic Acid | 291.121 | 0.62 | 2.47 | <0.001 |
|  | PC(22:4(7Z,10Z,13Z,16Z)/P-16:0) | 215.128 | 6.33 | 2.18 | <0.001 |
|  | N-Acetylglutamic acid | 505.251 | 2.94 | 2.14 | <0.001 |
|  | Serylglutamine | 489.184 | 2.70 | 1.96 | <0.001 |
|  | N-(alpha-Linolenoyl) Tyrosine | 366.130 | 2.85 | 1.91 | <0.001 |
|  | Uridine | 187.097 | 5.96 | 1.90 | <0.001 |
|  | Pyridoxine | 585.000 | 0.57 | 1.86 | <0.001 |
|  | Isoleucine | 259.130 | 2.93 | 1.85 | <0.001 |
|  | DL-Citrulline | 866.804 | 0.58 | 1.82 | <0.001 |
|  | Methylmalonic acid | 360.141 | 2.43 | 1.79 | <0.001 |
|  | Gamma-Glutamylarginine | 714.844 | 0.58 | 1.77 | <0.001 |
|  | 2-Hydroxyadenine | 420.920 | 0.54 | 1.75 | <0.001 |
|  | N-Acetylneuraminic acid | 227.103 | 3.15 | 1.75 | <0.001 |
|  | Serylthreonine | 277.086 | 2.66 | 1.74 | <0.001 |
|  | Isovalerylglutamic acid | 232.130 | 0.61 | 1.73 | <0.001 |
|  | Val-Ala-OH | 547.153 | 2.56 | 1.73 | <0.001 |
|  | Angelicolide | 712.997 | 0.56 | 1.72 | <0.001 |
|  | Alpha-Tetrasaccharide | 630.993 | 0.56 | 1.72 | <0.001 |
|  | Sonchuionoside C | 430.230 | 2.12 | 1.71 | <0.001 |
|  | Riesling acetal | 360.141 | 3.07 | 1.70 | <0.001 |
|  | Nomilinic acid 17-glucoside | 396.160 | 6.69 | 1.66 | <0.001 |
|  | Aspartyl-L-proline | 685.100 | 0.57 | 1.65 | <0.001 |
|  | Corchorifatty acid F | 537.901 | 0.58 | 1.65 | <0.001 |
|  | Urocanic acid | 241.119 | 4.26 | 1.65 | <0.001 |
|  | O-Cresol | 344.182 | 3.97 | 1.63 | <0.001 |
|  | Tyrosyl-Proline | 371.157 | 2.12 | 1.62 | <0.001 |
|  | Phenylalanyl-Valine | 304.115 | 4.26 | 1.62 | <0.001 |
|  | L-Glutamic Acid | 532.719 | 0.61 | 1.61 | <0.001 |
|  | Nalpha-Acetyl-L-glutamine N | 259.076 | 3.15 | 1.59 | <0.001 |
|  | L-cis-3-Amino-2-pyrrolidinecarboxylic acid | 474.760 | 0.61 | 1.57 | <0.001 |
|  | Delta-12-PGD2 | 656.886 | 0.58 | 1.57 | <0.001 |
|  | Prolylphenylalanine | 474.184 | 3.17 | 1.51 | <0.001 |
